# Causes of mortality in military working dog from traumatic injuries

**DOI:** 10.3389/fvets.2024.1360233

**Published:** 2024-07-08

**Authors:** Amanda P. Storer, Thomas H. Edwards, Christine R. Rutter, Grace E. Young, Sara B. Mullaney

**Affiliations:** ^1^School of Veterinary Medicine and Biomedical Sciences - Small Animal Clinical Sciences, Texas A&M University, College Station, TX, United States; ^2^US Army Institute of Surgical Research, Joint Base, Fort Sam Houston, TX, United States; ^3^United States Military Academy, Department of Chemistry and Life Science, West Point, NY, United States; ^4^Medical Center of Excellence, Division of Veterinary Science, Food Protection Branch, Joint Base San Antonio, Fort Sam Houston, TX, United States

**Keywords:** military working dog, mortality, head trauma, hemorrhage, canine, trauma, outcomes

## Abstract

**Introduction:**

This study aimed to identify the pathophysiologic causes of death following traumatic injuries in military working dogs (MWDs) and determine the risk factors associated with mortality in MWD following traumatic injuries. The results of this study will allow for better targeting of interventions to ameliorate these pathophysiologic causes of death and inform research priorities directed at the pathophysiology that leads to the death of MWDs.

**Methods:**

The final dataset for this study was compiled by using two previously established datasets. Based on review of available data and supplemental records (when available), MWDs in which a definitive cause of death could be determined were included in the study population. These MWDs were assigned a cause of death based on categories previously identified in studies evaluating service member casualties. A group of MWDs who survived their traumatic injury and had similar mechanisms of injury and types of injury to the deceased MWDs were included to allow for comparison and establishment of risk factors associated with MWD death. Variables collected included breed, age, sex, mechanism of injury, survival/non-survival, type of trauma, mechanism of injury, pathophysiology that led to death and pre-hospital care provided. Statistical analysis included Fishers exact test for categorical variables and univariable and multivariable logistic regression to identify factors associated with the MWD death.

**Results:**

A total of 84 MWDs (33 non-survivors and 51 survivors) were included in this study. Of the 33 MWDs that died, 27 (81.8%) were noted to be dead on arrival. The pathophysiologic causes of death were found to be hemorrhage (45.5% [*n* = 15]), head trauma (21.2% [*n* = 7]), catastrophic tissue destruction (15.2% [n = 5]), pneumothorax (9.1% [*n* = 3]) and one (3% [*n* = 1]) of each of the following: septic shock, asphyxiation and burns. Military working dogs that did not receive non-DVM care were 3.55 times more likely to die than those that did receive non-DVM care (95% CI 1.03–12.27). The majority of MWDs died of their injuries before reaching veterinary care.

**Discussion:**

To increase the survival of MWDs on the battlefield, further research should focus on developing new interventions and techniques to mitigate the effects of the pathophysiology noted to cause MWD death. Furthermore, given that care by a non-DVM was found to be associated with survival, the implementation of pre-hospital care and early resuscitation techniques should be a continued priority for those treating MWDs at both the point of injury and in the prehospital setting.

## Introduction

Military working dogs (MWDs) have served alongside service members on the battlefield since ancient times. Their primary duty is the detection of explosives and improvised explosive devices (IEDs) and deterring enemy threats (1–4). These tasks have undoubtedly saved countless lives on the battlefield and their innate capability to detect explosives far exceeds any technologies available today (5). While this work has prevented catastrophic injuries to service members, it does not come without risks to the MWDs (1, 2, 6). Military working dogs are often exposed to various threats during deployment, putting them at risk of sustaining a serious traumatic injury during their service (1–4, 6).

Traumatic injury is a common cause of mortality in civilian dogs, operational canines, and MWDs (1). A study evaluating morbidity and mortality in civilian dogs, showed that trauma is the second leading cause of death in both juvenile and adult civilian dogs (7). In deployed MWDs, a mortality study found that 77% of deaths were due to traumatic injuries with IEDs and guns shot wounds (GSW) being the leading causes of both injury and death (3). Traumatic injury is not limited to dogs; it also occurs in both military and civilian populations and it is the leading cause of death in people (ages 10–44) in the United States (8–10). Traumatic death as seen on the battlefield and lessons learned from wounded service members can be used to improve the care of injured people everywhere (9, 10).

The alterations in physiology caused by trauma that led to mortality in people and animals are varied. A landmark study on service members killed in the recent wars in Iraq and Afghanistan identified hemorrhage, upper airway obstruction and pneumothorax as the leading causes of death (9). In another study on special operations forces personnel killed in these conflicts, catastrophic tissue destruction was found to be the leading mechanism of death (10). The identification of the leading causes of death in service members has directly driven funding into biomedical research to counter the pathophysiologies that lead to these deaths. This research has led to development of biomedical innovations such as advanced tourniquets, hemostatic dressings, and improved policies on blood transfusions (11–13). One study found that approximately 1,300 service members were saved and there was a 44.2% reduction in overall mortality because of the employment of the innovations developed to address life threatening hemorrhage; the leading cause of death in the conflicts reviewed (14).

To date, there is no such analysis of the causes of traumatic death in MWDs. If the causes of death from trauma can be identified in MWDs, funding can be directed toward developing new devices and techniques aimed at preventing MWD traumatic death. Despite the mechanisms of injury (MOIs) and trauma types differing between MWDs and civilian dogs (1), understanding the causes of death could not only be beneficial for injured MWDs but also injured civilian dogs. To the authors knowledge, there are no studies that identify the pathophysiologic causes of death in the civilian dog population. The lack of studies identifying the pathophysiologic causes of death in the civilian dog population may be related in part to euthanasia bias in civilian dogs which is not present in MWDs. While MWDs may be euthanized for various reasons, including poor prognosis and to alleviate suffering, they are not euthanized as a result of financial constraints. We therefore undertook this study in MWDs who have minimal to no euthanasia bias.

The primary objective of this study was to identify the pathophysiologic causes of death following traumatic injuries in MWDs. A secondary objective was to determine the risk factors associated with mortality in MWD following traumatic injuries. We hypothesized that the causes of death in MWDs would closely reflect the causes of death previously identified in service members, with hemorrhage being the leading cause of death. We also expected that care by a non-DVM in the pre-hospital setting would be associated with MWD survival.

## Materials and methods

### Study population

**Figure 1:**
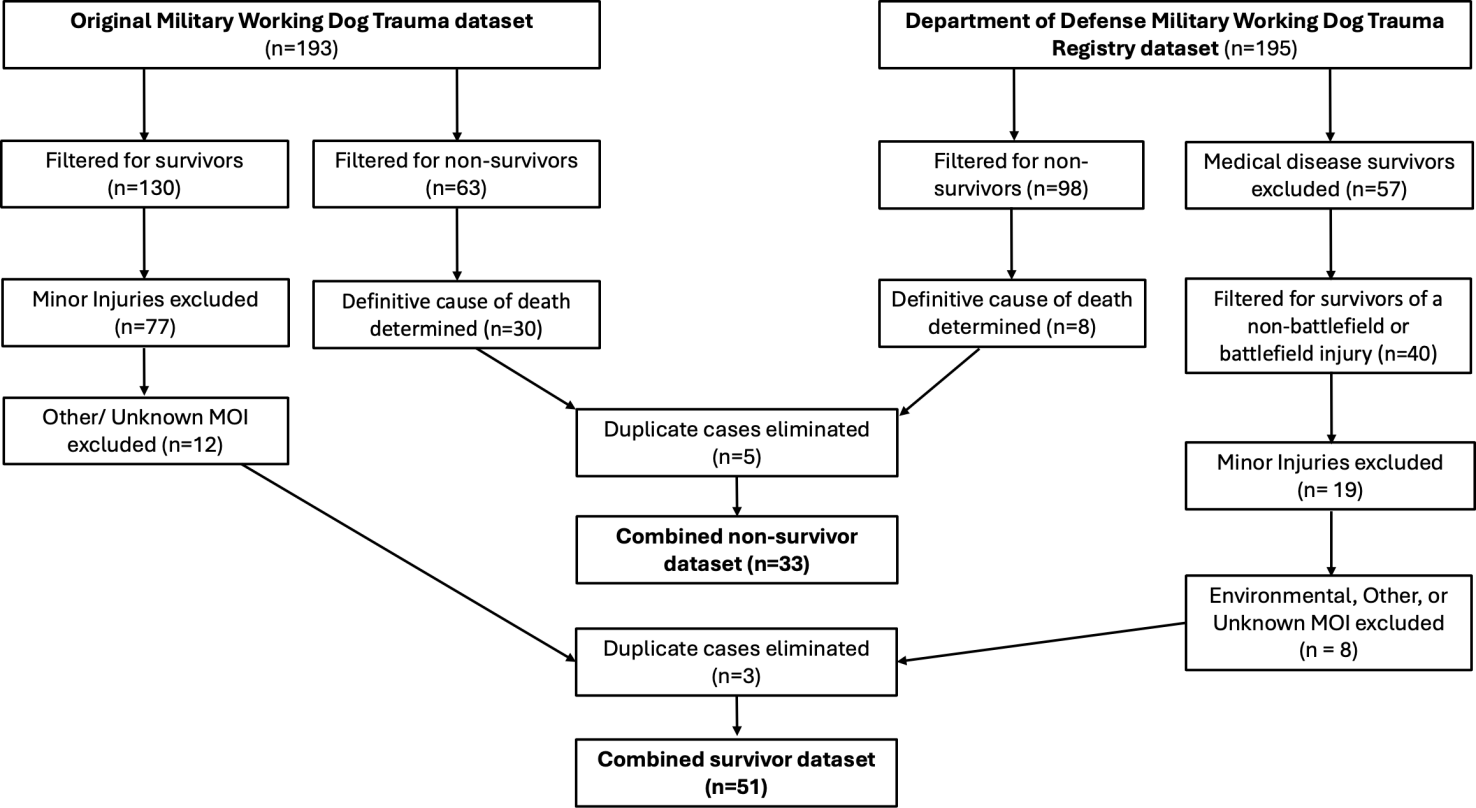
Methodology for inclusion/exclusion of military working dogs.

The study population of non-survivors and survivors is a compilation of two previously established datasets (Figure 1). The first dataset included data from a study on MWD trauma (1). This dataset included 165 United States (US) MWDs that sustained traumatic injuries, with some MWDs experiencing more than one injury for a total of 193 injury events during their deployment within the Central Command’s area of responsibility from September 11, 2001 to December 31, 2018 (1). The second dataset included data from the recently established Department of Defense (DoD) MWD trauma registry. This dataset included 141 US MWDs with documented veterinary visits between 2006 and 2023 for varying conditions including ongoing disease management, non-battlefield injuries and battle-field injuries. Some of the MWDs had more than one documented veterinary visit for a total of 195 veterinary visits.

### Non-survivors

To establish a set of non-survivors for analysis from the MWD trauma dataset (1), the dataset was filtered for MWDs that were noted to have died following their injury event, which included 63 injury events to be reviewed by the authors. To supplement the information available in the dataset, official veterinary treatment records (VTRs; including, but not limited to, health records, necropsy reports and death certificates), were reviewed for additional information such as description of injury, diagnosis, and necropsy findings. After this information was collected, the non-survivor MWD data was reviewed by all authors to determine if a definitive cause of death could be identified. To determine cause of death, the authors used a combination of necropsy findings (*n* = 15), description of injury (ex. GSW to head in combination with immediate death, lack of remains following injury, etc.), treatments performed and any diagnosis from medical personnel (point of injury) or veterinarians attending to the MWDs. If the authors did not agree on a definitive cause of death, the MWD was excluded from the study population. For all non-surviving MWDs with an available necropsy, a pathophysiologic cause of death was able to be determined. If a definitive cause of death was determined (*n* = 30) the MWD met inclusion criteria and a cause of death was classified based on categories previously identified for service members (9, 10). The categories for cause of death included asphyxiation, burns, catastrophic tissue destruction, head trauma, hemorrhage, pneumothorax, and septic shock.

Similar to the first dataset, the DoD MWD trauma registry was filtered for MWDs that were noted to have died, which resulted in 98 deaths to be reviewed by the authors. Military working dogs that died or were euthanized secondary to diagnosed medical conditions (disease not related to battlefield injuries), behavioral reasons, quality of life concerns and unknown reasons were excluded. Of the deaths remaining, if a definitive cause of death could not be determined the MWD was also excluded. If a definitive cause of death was determined (*n* = 8) the MWD met inclusion criteria and a cause of death was classified based on categories as discussed previously. Given that some MWDs were contained in both the MWD trauma dataset (1) and the DoD MWD trauma registry, records were screened and duplicate injury events were eliminated for the final non-survivor dataset.

### Survivors

A subset of survivors from the previously mentioned datasets, were selected by identifying MWDs that sustained a traumatic injury secondary to a mechanism of injury that had the potential to cause lethal injury. These mechanisms of injury included: explosive events, gunshot wounds (GSW), shrapnel, fall, laceration, burn, struck by vehicle, electrocution, smoke inhalation, kicked by cow and crush injury.

To develop a list of survivors from the MWD trauma dataset (1), the dataset was filtered for MWDs that were noted to survive to discharge following their injury event (*n* = 130). Similar to the non-survivors, hard copies of available VTRs were reviewed for additional information such as description of injury, physical exam findings and diagnosis to supplement the information available in the dataset. After this information was collected, the survivor MWD data was reviewed by the authors. Military working dogs were excluded from analysis if injuries were noted to be minor injuries (ex. tail wounds, torn nail/paw pads, superficial lacerations, falling from entering or exiting a vehicle, minor animal bites/scratches, etc.) that allowed the MWD to be treated on an outpatient basis and would not limit the MWDs ability to continue deployment responsibilities. Further exclusion criteria included MWDs noted to have other or unknown mechanisms of injury. Inclusion criteria included MWDs that sustained injuries that had the potential to cause lethal injury including injuries secondary to gunshots, IEDs and or other explosions and injuries that could not be amenable to outpatient care alone (ex fractures, dislocated joints, etc.).

A similar process was completed with the DoD MWD trauma registry dataset to develop a list of MWDs who survived to be reviewed for inclusion. The dataset was filtered for MWDs who sustained injuries in either a non-battlefield or battlefield setting and were noted to survive to discharge following their injury event, which resulted in 40 injury events to be reviewed. As for the MWD trauma registry dataset, exclusion criteria included minor injuries (ex. tail wounds, torn nail/paw pads, superficial lacerations, falling from entering or exiting a vehicle, animal bites/scratches, etc.) that allowed the MWD to be treated on an outpatient basis and did not limit the MWDs ability to continue deployment responsibilities. Military working dogs were further excluded from analysis if the mechanism of injury was noted to be environmental, other, or unknown. Inclusion criteria was the same as that for the MWD trauma registry dataset. Similar methodology was used to eliminate duplicate injury events from the final datasets.

### Data

Data abstraction and details regarding classification of key demographic and injury characteristics were used as previously described (1). Data from the DoD MWD trauma registry was used as reported in the registry. Prehospital care was examined based on whether care was provided (yes, no, unknown), the type of care provided (none, bandage and wound flush, multiple resuscitation procedures, unknown, other) and the non-DVM care provider (none, unknown, two or more personnel, unspecified military personnel, medic, handler, physician or other human healthcare provider) for both outcomes. The variables for the type of care provided and the non-DVM care provider, were created based on the information provided in the datasets.

For type of care provided, if no care was provided the variable “none” was assigned; if the type care provided was not specified the variable “unknown” was assigned; if the type of care provided was a specified the type of care was assigned. Military working dogs were considered to have had multiple resuscitation procedures if they received two or more interventions. Military working dogs with multiple interventions included the following interventions: intraosseous catheters, chest seals, tranexamic acid, hemostatic agents, drugs for sedation, resuscitation, venous cut downs, bandages, wound flushing needle decompression and emergent tracheostomy.

In regards to non-DVM care provider, if no care was provided the variable “none” was assigned, if care was noted to be provided by someone and not specified the variable “unknown” was assigned, if care was provided by the handler or owner the variable “handler” was assigned, if care was provided by a combat medic, EMT, police or firefighter the variable “medic” was assigned, if care was provided by a military personnel the variable “unspecified military personnel” was assigned, and finally if care was provided by a physician or nurse the variable “physician/other human healthcare provider” was assigned. The non-DVM care provider was considered to be two or more providers if the MWD was treated by two or more different types of providers (ex. EMT and handler, medical doctor and handler, etc.).

Military working dogs were classified as having experienced an immediate death if they were found to be dead on arrival (DOA) to veterinary care. They were classified as having experienced an early death if they survived to admission to the medical or veterinary treatment facility and died within 48 h of arrival. Military working dogs were classified as having experienced late death if they died after this time.

Anatomic location of injury was characterized by the site on the MWD body where the primary injury occurred. Body regions used were selected based on previous canine injury research (1, 32). The location of injury was characterized as unspecified if more than one location could have been affected (ex. a MWD in close contact with an IED or explosion and presented for shaking of the head following the explosion) and a definitive location of injury was not obvious from the available records. Catastrophic tissue destruction was defined as serious injuries to multiple anatomic locations with immediate death or lack of identifiable remains after an IED injury.

### Statistical analysis

Data was compiled into to commercially available spreadsheet for aggregation, filtering, and creation of figures (Microsoft Excel version 16.76. Redmund, WA). Descriptive statistics for categorical variables included frequency, percentages, and relative 95% confidence intervals (CI). Differences in proportion were assessed by the Fisher’s exact test. The continuous variable (age) was described by the histogram, mean and standard deviation, or median and range. Differences in median age were assessed by a Wilcoxon Signed Rank Test. Univariable and multivariable logistic regression were used to identify factors associated with the MWD death. The explanatory variables included sex (male intact, female spayed, male castrated), breed (Belgian Malinois, German Shepherd, Labrador Retriever, other, unknown), type of trauma (blunt and penetrating; penetrating; burn, electrical, or chemical; blunt), Non-DVM care Provided (yes, no, unknown), and type of care provided (multiple resuscitation procedures, bandage and/or wound flush, none, other, unknown). In the multivariable analysis, all variables with *p*-value <0.25 from the univariable analysis were included (sex, type of trauma, non-DVM care provided, and type of care provided). These variables were subsequently removed in a step-wise fashion beginning with the highest *p*-value until all variables were p-value >0.05 were removed. Final model fit was assessed using the Hosmer Lemeshow Goodness of Fit Test, with *p* > 0.05 indicating goodness of fit. All statistical analysis was performed using SAS® Studio in SAS ® OnDemand for Academics (2023 SAS Institute Inc.).

## Results

A total of 388 separate MWD veterinary events between 2001 and 2023 were evaluated for inclusion in the study population (Figure 1). Of these veterinary events, 161 resulted in MWD death. Military working dogs that died were excluded from the non-survivor study population if they died or were euthanized secondary to diagnosed medical conditions (disease not related to battlefield injuries; *n* = 61), behavioral reasons (*n* = 14), quality of life concerns (*n* = 4) and unknown reasons (*n* = 5), if a definitive cause of death could not be determined (*n* = 39) and if they were duplicates within the databases (*n* = 5). Of these veterinary events, 227 resulted in MWD survival. Military working dogs that survived were excluded from the survivor study population if their mechanisms of injury were noted to be any of the following: environmental (*n* = 4), other (*n* = 2) or unknown (*n* = 14). Military working dogs were further excluded if they survived a medical condition that did not lead to injury (ex gastro-intestinal upset; *n* = 57), if they sustained a minor injury (ex. tail wounds, torn nail/paw pads, superficial lacerations, falling from entering or exiting a vehicle, animal bites/scratches, etc.; *n* = 96) and if they were duplicates within the databases (*n* = 3). See Figure 1.

A total of 84 MWDs met the inclusion criteria including 33 non-survivors and 51 survivors. Of the 33 non-survivors, 81.8% were noted to be DOA (and therefore classified as immediate deaths) and the remainder 18.2% died during hospitalization (classified as early deaths); none of the deceased MWDs were euthanized as a result of their injuries. There were no late non-survivors included in the study.

Table 1 displays MWD demographic and injury characteristics by outcome. The age variable was not normally distributed and is displayed as median and range. Majority of the population were male and intact (66.7% [*n* = 56]); of which 28 (54.9%) were survivors and 28 (84.9%) were non-survivors. Regarding trauma type, the majority of injuries were penetrating in nature for both survivors (54.9% [*n* = 28]) and non-survivors (54.6% [*n* = 18]). The most common mechanism of injury (MOI) for the entire study population was an explosive event (34.5% [*n* = 29]). For survivors, the most common MOI was also explosive events (35.3% [*n* = 18]), whereas for the non-survivors the main MOI was gunshot wound (GSW; 45.5% [*n* = 15]) followed by explosive events (33.3% [*n* = 11]), strike by vehicle (6.1% [*n* = 2]) and one (3% [*n* = 1]) of each of the following burns, electrocution, laceration, smoke inhalation, and simultaneous GSW and grenade.

**Table 1:** Military working dog demographics and injury characteristics by outcome (Survivors/Non-survivors).

Characteristic	Total	Outcome		*p*-value
	*N* = 84	Survivors	Non-survivors	
		*n* = 51	*n* = 33	
Age in years (median, range)^*^	4.5 (1.5–9.0)	4.5 (1.5–8.5)	5.8 (1.9–9.0)	0.134^#^
Sex	n (%)	n (%)	n (%)	0.020^#^
Male, intact	56 (66.7)	28 (54.9)	28 (84.9)	
Male, castrated	18 (21.4)	15 (29.4)	3 (9.1)	
Female, spayed	10 (11.9)	8 (15.7)	2 (6.1)	
Breed	n (%)	n (%)	n (%)	0.544^**^
Belgian Malinois	43 (51.2)	23 (45.1)	20 (60.6)	
German Shepherd	22 (26.2)	16 (31.4)	6 (18.2)	
Labrador Retriever	9 (10.7)	5 (9.8)	4 (12.1)	
Other^†^	5 (6.0)	3 (5.9)	2 (6.1)	
Unknown	5 (6.0)	4 (7.8)	1 (3.0)	
Type of Trauma	n (%)	n (%)	n (%)	0.001^**^
Penetrating	46 (54.8)	28 (54.9)	18 (54.6)	
Blunt	19 (22.6)	17 (33.3)	2 (6.1)	
Blunt and Penetrating	16 (19.1)	6 (11.8)	10 (30.3)	
Burn, Electrical or Chemical	3 (3.6)	0 (0.0)	3 (9.1)	
Mechanism of Injury (MOI)	n (%)	n (%)	n (%)	0.003^**^
Explosive Event^‡^	29 (34.5)	18 (35.3)	11 (33.3)	
Gun Shot Wound	27 (32.1)	12 (23.5)	15 (45.5)	
Shrapnel	9 (10.7)	9 (17.7)	0 (0.0)	
Fall	6 (7.1)	6 (11.8)	0 (0.0)	
Laceration	4 (4.8)	3 (5.9)	1 (3.0)	
Other ^¶^	9 (10.7)	3 (5.9)	6 (18.2)	

*Age data missing for three military working dogs. ^†^Other Breed category includes long/short-haired Dutch Shepherd, Belgian Tervuren, and English Springer Spaniel. ^‡^Explosive event includes grenade, improvised explosive device, and other explosion. ^¶^Other Mechanism of Injury category includes burn, struck by vehicle, electrocution, gunshot wound and grenade, smoke inhalation, kicked by cow and crush injury. ^#^Median two-sample nondirectional test where the null hypothesis median age of survivors and non-survivors is the same. **Fisher’s exact test where the null hypothesis is there is no difference between groups.

Prehospital care and its association with outcome for trauma among MWDs is presented in Table 2. Overall, the majority (69.1% [*n* = 58]) of the population had no documented care prior to admission to a veterinary hospital. Thirty-one (60.8%) of the survivors did not receive pre-hospital care from a non-veterinarian, while 27 (81.8%) of the non-survivors did not receive pre-hospital care from a non-veterinarian. Of the subpopulation that received pre-hospital care, the majority of the care administered were bandages or wound flush (16.7% [*n* = 14]), with most being delivered to survivors (25.5% [*n* = 13]). Of the six non-survivors who received care 50% [*n* = 3] required multiple resuscitative procedures. The most common prehospital treatment was delivered by two or more providers for the total population (9.5% [*n* = 8]) and for the survivors (13.7% [*n* = 7]). For non-survivors, the majority received no prehospital care. Of the six non-survivors who did receive care, 33% [*n* = 2] received care from the handler whereas the other four MWDs received care from a physician or other human healthcare provider [*n* = 1], two or more providers [*n* = 1], unspecified military personnel [*n* = 1], and medic [*n* = 1]. The type of care provided (*p = 0.003*) was the only significant variable in predicting the outcome. Non-DVM care provided (*p = 0.09*) and the non-DVM care provider (*p = 0.114*) was not significantly associated with predicting the survival of MWDs.

**Table 2:** Pre-hospital care associated with outcome (Survivors/Non survivors) for military working dogs experiencing traumatic injuries.

	Total	Outcome		
	*N* = 84	Survivors	Non-survivors	
		*n* = 51	*n* = 33	*p*-value^‡^
Non-DVM Care Provided	n (%)	n (%)	n (%)	0.09
No	58 (69.1)	31 (60.8)	27 (81.8)	
Yes	24 (28.6)	18 (35.3)	6 (18.2)	
Unknown*	2 (2.4)	2 (3.9)	0 (0.0)	
Type of Care Provided	n (%)	n (%)	n (%)	0.003
None	58 (69.1)	31 (60.8)	27 (81.8)	
Bandage &/or Wound Flush	14 (16.7)	13 (25.5)	1 (3.0)	
Unknown*	8 (9.5)	6 (11.8)	2 (6.1)	
Multiple Resuscitation Procedures	3 (3.6)	0 (0.0)	3 (9.1)	
Other^†^	1 (1.2)	1 (2.0)	0 (0.0)	
Non-DVM Care Provider	n (%)	n (%)	n (%)	0.114
None	58 (69.1)	31 (60.8)	27 (81.8)	
Unknown*	6 (7.1)	6 (11.8)	0 (0.0)	
Two or More Providers	8 (9.5)	7 (13.7)	1 (3.0)	
Unspecified Military Personnel	4 (4.8)	3 (5.9)	1 (3.0)	
Medic	3 (3.6)	2 (3.9)	1 (3.0)	
Handler	3 (3.6)	1 (2.0)	2 (6.1)	
Physician or other human healthcare provider	2 (2.4)	1 (2.0)	1 (3.0)	

*Data was not available in the veterinary records. ^†^Other care provided category includes oral medication administration. ^‡^Fisher’s exact test where the null hypothesis is there is no difference between groups.

**Figure 2:**
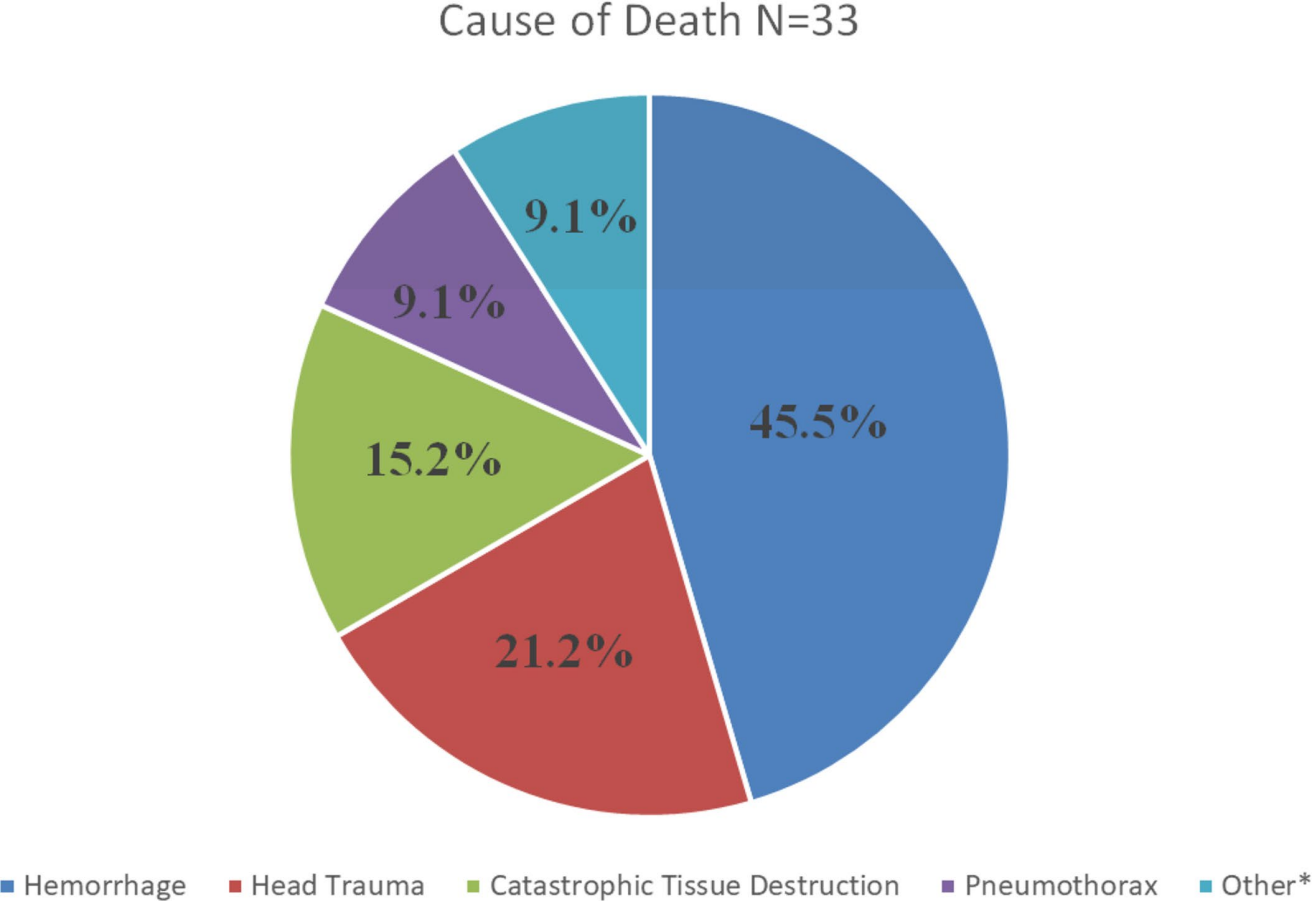
Pathophysiologic causes of traumatic death in MWDs. *Other includes septic shock, asphyxiation and burns.

**Figure 3:**
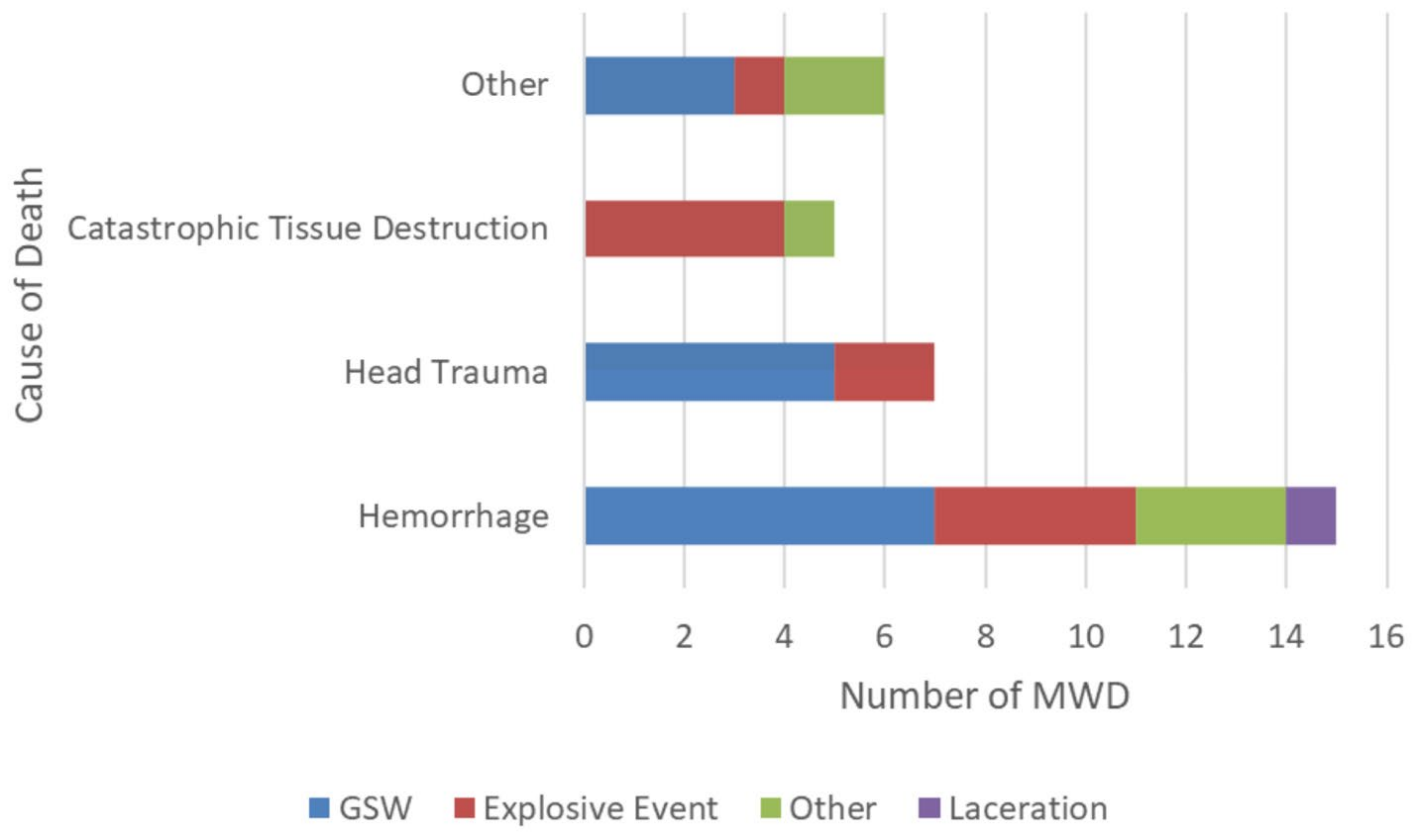
Pathophysiologic causes of death by mechanism of injury.

The pathophysiologic causes of death in MWDs were hemorrhage (45.5% [*n* = 15]), followed by head trauma (21.2% [*n* = 7]), catastrophic tissue destruction (15.2% [*n* = 5]), pneumothorax (9.1% [*n* = 3]), and one each (3% [*n* = 1]) of septic shock, asphyxiation, and burns (Figure 2). Of the MWDs that died due to hemorrhage, the most common MOI was GSW (46.6% [*n* = 7]) and explosive events (26.6% [*n* = 4]; Figure 3). For the MWDs that died due to head trauma, the main MOI was GSW (71.4% [*n* = 5]). Of the MWDs that died due to catastrophic tissue destruction, explosive events (80% [*n* = 4]) were the main MOI.

The anatomical location of injury by outcome is presented in Table 3. The majority of MWDs experience injury to the limb (40.5% [*n* = 17]) and in an unspecified location (40.5% [*n* = 17]). For non-survivors, the majority experienced injury to multiple locations (35.7% [*n* = 10]). The location of injury (*p = 0.0001*) was determined to be significant in predicting the outcome for MWDs.

**Table 3:** Anatomic location of injury and association with outcome in military working dogs.

	Total	Outcome		
	*N* = 70*	Survivors	Non-survivors	
		*n* = 42	*n* = 28	*p*-value^¶^
Location of Injury	n (%)	n (%)	n (%)	0.0001
Limb	19 (27.1)	17 (40.5)	2 (7.1)	
Unspecified^†^	17 (24.3)	17 (40.5)	0 (0.0)	
Head	12 (17.1)	3 (7.1)	9 (32.1)	
Multiple^‡^	12 (17.1)	2 (4.8)	10 (35.7)	
Thorax	8 (11.4)	3 (7.1)	5 (17.9)	
Abdomen	2 (2.9)	0 (0.0)	2 (7.1)	

*Animals experiencing burns, electrocution, falls, smoke inhalation, struck by vehicle, kicked by cow, and crush injury as their MOI were excluded from analysis (*n* = 14). ^†^Definitive location could not be determined based on available in the veterinary records. ^‡^Two or more anatomical locations (ex. thorax and abdomen, thorax and limb, etc.). ^¶^Fisher’s exact test where the null hypothesis is there is no difference between groups.

Univariable and multivariable logistic regression analysis results are presented in Table 4. In the univariable analysis, breed, type of trauma, and type of care provided were associated with death. In the multivariable model, sex, type of trauma, and non-DVM care provided were associated with death. Male intact MWDs with blunt and penetrating trauma or penetrating trauma (only) that received no medical care prior to presentation to a DVM were significantly more likely to die. When controlling for sex and receipt of care from non-DVM, dogs that experienced blunt and penetrating trauma were 13.38 times more likely to die than those who experienced only blunt trauma (95% CI: 2.02–88.57). When controlling for sex and receipt of care from non-DVM, dogs that experienced penetrating trauma (only) were 7.72 times more likely to die than those who experienced only blunt trauma (95% CI: 1.40–42.73). When controlling for receipt of care from non-DVM and trauma type, male intact MWDs were 7.64 times more likely to die than male castrated MWDs (95% CI: 1.44–40.39). When controlling for sex and trauma type, MWDs that did not receive non-DVM care were 3.55 times more likely to die than those that did receive non-DVM care (95% CI 1.03–12.27).

**Table 4:** Univariable and multivariable logistical regression analysis of risk factors associated with military working dog outcome.

	Univariable analysis			Multivariable analysis		
	Odds ratio	95% CI	*p*-value	Odds ratio	95% CI	*p*-value
Sex						
Male, intact	**5.0**	**(1.32–19.20)**	**0.019**	**7.64**	**(1.44–40.39)**	**0.017**
Female, spayed	1.25	(0.17–9.09)	0.826	2.68	(0.27–26.76)	0.400
Male, castrated	REF			REF		
Breed						
Belgian Malinois	3.48	(0.36–33.7)	0.282			
German Shepherd	1.5	(0.14–16.3)	0.739			
Labrador Retriever	3.2	(0.25–41.2)	0.372			
Other^†^	2.7	(0.16–45.1)	0.468			
Unknown	REF					
Type of Trauma						
Blunt and Penetrating	**14.17**	**(2.39–84.1)**	**0.0035**	**13.38**	**(2.02–88.57)**	**0.007**
Penetrating	**5.46**	**(1.13–26.5)**	**0.0352**	**7.72**	**(1.40–42.73)**	**0.019**
Burn, Electrical or Chemical	***			***		
Blunt	REF			REF		
Non-DVM Care Provided						
No	2.613	(0.907–7.53)	0.075	**3.55**	**(1.03–12.27)**	**0.045**
Unknown	***					
Yes	REF					
Type of Care Provided	***					
Multiple Resuscitation Procedures	***					
None	**11.32**	**(1.39–92.31)**	**0.0234**			
Other	***					
Unknown	4.33	(0.33–57.65)	0.2668			
Bandage &/or Wound Flush	***					

Bold indicates statistically significant values (*p* value < 0.05). ***Indicates quasi-complete separation of data points due to sparse cells.

## Discussion

This is the first study to report on the pathophysiologic causes of death in dogs from trauma and more specifically MWDs. While previous studies (1, 3) have reported mechanisms of injury resulting in MWD death, none have described the pathophysiology that leads to death. In 2015, Eastridge et al. identified the most common pathophysiologies leading to battlefield deaths among military service members (9). This work led to research and development efforts aimed at mitigating the responsible pathophysiologies (hemorrhage, airway obstruction, and tension pneumothorax). These mitigation strategies have resulted in a reduction of US military combat causalities by an impressive 44.2% (14). Although MWDs have a similar risk of trauma and death on the battlefield as service members, to date, there is less funding allocated to casualty care research for MWDs (15). While advances in care have reduced service member causalities on the battlefield, these treatments are not always directly applicable in dogs given the physiologic and anatomic differences between dogs and humans (11, 15, 16). By identifying and understanding the pathophysiologic causes of battlefield death in MWDs, similar research efforts and funding can be focused toward developing new modern interventions and techniques to mitigate the effects of the pathophysiology noted to cause MWD traumatic death, most notably hemorrhage and head trauma.

It is well known that hemorrhage is a leading cause of death in both military service members and civilian trauma cases (9, 17). In this study, hemorrhage was the leading cause of traumatic death among MWDs, with just under half of the MWDs dying secondary to acute hemorrhage (Figure 2). Although the percentage of MWDs that died secondary to hemorrhage is not as high as reported in service member causalities (90.9%) (9), it is not surprising that hemorrhage was identified as the leading cause of death in our MWD population given the predominant mechanisms of injury are well-known to cause hemorrhage (3). To mitigate the pathophysiologic consequences associated with hemorrhagic shock, timely hemorrhage control along with appropriate replacement of blood volume is necessary (11, 12, 18). A recent publication, reviewed the advancements made in human medicine to mitigate pre-hospital hemorrhage and discussed how these advancements can be translated to injured dogs (11). While some advancements such as hemostatic dressings, hemostatic devices and certain limb tourniquets (Stretch, Wrap, And Tuck-Tourniquet) can be used and applied to injured MWDs, there is a lack of evidence evaluating the application and effectiveness of these products in veterinary patients (11). Given that canine blood products can be a challenge to resource and deliver in the pre-hospital setting, innovations that can help reduce the risk of MWD death from hemorrhage should be a research priority (5). The US military is currently working on a number of initiatives in this area to include fielding canine whole blood on the battlefield as well as research into shelf stable blood products and blood substitutes such as hemoglobin based oxygen carriers (HBCOs) and canine freeze-dried plasma (cFDP) (12). A previous study evaluating the hemostatic capacity of cFDP and HBOC in an *in vitro* model of resuscitation showed promising results and concluded that additional research is warranted to determine if cFDP reconstituted with HBOC is a viable resuscitation product in canine trauma (19). Future research should focus on evaluating and potentially deploying these or similar products into the prehospital setting for MWDs.

Head trauma was the second leading cause of death in this population of MWDs. This is in contrast with the study by Eastridge et al. (9), the aforementioned study in service members, who found airway obstruction and tension pneumothorax were the second and third most common pathophysiology that led to death. This also differs from the study by Mazuchowski and colleagues (10) who found that catastrophic tissue destruction and hemorrhage were the two most common causes of death in special operations service members during the recent conflicts which took place in Afghanistan and Iraq. There are several explanations for the differences found in this study on dogs and the two aforementioned studies in people. Service members typically deploy with state-of-the-art helmets which are specially designed to mitigate head injuries. Military working dogs are not currently afforded helmets which may explain why head injuries resulted in death in these MWDs. In the present study, in non-surviving MWDs, head injuries most often were from high velocity injuries. Because service members typically wear ballistic helmets in combat, many of these head injuries, while often severe, are not as catastrophic since the helmet absorbs a portion of the force. Secondly, non-catastrophic head injuries are more easily screened in people who are able to understand commands and communicate injuries as opposed to canine patients, potentially allowing for earlier diagnosis and intervention in people. Finally, treatment recommendations are more refined in people by severity with head injuries than they are in dogs, potentially leading to more consistent treatments and improved outcomes (20, 21).

Gunshot wounds were not only the most common MOI leading to death, but also the most common MOI leading to acute hemorrhage and head trauma. Explosive events were the second most common MOI leading to death. These findings are consistent with a previous study that evaluated the MOI leading to death in MWDs, with the GSW and explosion events being responsible for 31.5 and 26.1% of traumatic injuries resulting in death, respectively (3). The proportion of battlefield deaths related to GSW and explosive events in our study and previous studies is consistent with the high-risk task that MWDs perform in their line of duty (1–4). GSW and explosive events are also the most common MOIs leading to injury in service members (22), further supporting that MWDs are exposed to similar risks as service members.

The high prevalence of penetrating injuries among MWDs in the study population is not surprising considering that most of the MOIs (GSW, lacerations, and shrapnel from explosive devices) in these dogs are known to cause penetrating injuries, with explosions often causing both blunt and penetrating injuries. The multivariable analysis showed that MWDs that experienced blunt and penetrating trauma or penetrating trauma alone were at increased risk of death compared to MWDs that experienced only blunt trauma. While never assessed in previous studies on MWD trauma, this finding reflects the result of a recent study in civilian dogs which found that dogs suffering from both penetrating and blunt injury experienced moderate to severe injury, lower survival rates, and were more likely to be admitted to the ICU compared to patients suffering either penetrating or blunt injury (23). In contrast to our findings of penetrating trauma leading to an increased risk of death in MWDs compared to blunt trauma, civilian dogs that experienced penetrating trauma had a better survival rate (96.5%) compared to those that experienced blunt trauma (89.5%) (23). The difference within these two populations of dogs is likely a result of the predominant MOIs causing trauma in these populations. In civilian dogs, penetrating trauma is most common a result of bite wounds (71.3%) with ballistics (0.9%), impalements (1.9%) and lacerations (7.7%) occurring less frequently (24); whereas in MWDs, GSWs and shrapnel are the predominant MOI which can be severe and fatal.

When comparing non-survivors to survivors, the majority of non-survivors experienced their traumatic injury to either multiple anatomic locations (i.e., thorax and abdomen, thorax and limb, etc.), the head and or the thorax whereas the majority of survivors experienced their traumatic injury to a limb. A study by Baker et al. (6) which described GSW injuries in MWDs found that while location of wounds was not significantly associated with outcome, all MWDs in the study population that received wounds to the neck or abdomen died of their wounds, suggesting that GSW to the neck and abdomen tend to cause fatal trauma. This study also found that MWDs with extremity wounds were more likely to survive compared to dogs with other injury locations (6). In service members, musculoskeletal extremity injuries have not only been reported to comprise approximately 50% of all combat wounds, they also have been found to be one of the common causes of death secondary to hemorrhage (25–27). The difference between canine and human extremity injuries in regards to hemorrhage control and blood loss, might be explained by the difference in muscle mass, with dogs having relatively less than humans. This may result in less hemorrhage and allow for easier compression of damaged blood vessels; with canine hemorrhage often being controlled with the application of direct pressure and pressure bandage and rarely warranting tourniquet application (11). Thoracic injuries in service members have decreased from 33% to. 4.6%, secondary to the use of individual body armor (25). The use of body armor provides protection to the head, thorax and abdomen and thus reduces the overall percentage of thoracic injuries and diminishes the impact of what might otherwise be life-threatening injuries (25). While body armor is available for MWDs, there are several limitations to its use including that it is often not well tolerated by the MWD, it does not have the same ballistic rating as human body armor and it is thought to contribute to fatigue and heat injury (2, 6).

Of the 33 non-survivors, 81.8% died before reaching veterinary care. This finding is similar to service members and civilian trauma cases, with approximately 40–87% of post-traumatic casualties dying in the pre-hospital period, e.g., before reaching a medical treatment facility (9, 26–30). Death after trauma occurs in three phases known as the immediate, early and late phase, respectively, (31). Immediate deaths tend to occur in those who suffer an overwhelming and catastrophic injury, such as close proximity to an explosive device. Early deaths tend to occur within minutes to hours of the injury and are often the result of hemorrhage, respiratory compromise or traumatic brain injury, whereas late deaths often occur secondary to organ dysfunction (31). The data presented here in MWDs shows a similar pattern to service members, in so far as the majority of deaths in both groups occurred in the immediate phase followed by the early phase and the fewest in the late phase. In service members, many pre-hospital fatalities occur within minutes of the injury and are classified as deaths that could be prevented through early implementation of resuscitation techniques (26, 28, 31) One of the most influential developments for reducing fatality rates and advancing pre-hospital casualty care in service members has been the development of Tactical Combat Casualty Care (TCCC) guidelines (17, 31, 32, 33). Given that veterinary personnel are not typically present at the point of injury, pre-hospital care for MWDs that sustain combat-related trauma is often provided by either the MWD handler or non-veterinary healthcare providers (i.e., medics, corpsmen, nurses, midlevel providers, and physicians) (1, 2, 6). In part, as a result of the success of TCCC in reducing fatalities in service members, canine-specific TCCC guidelines have been developed; with the most recent version published in 2023 (34). Based on the causes of death determined in this study, it is likely that most of the MWDs who died experienced either immediate or early deaths secondary to their injuries. Regardless of when the death occurred, our study suggests that MWDs who do not receive non-DVM care are more likely to die than those that do receive non-DVM care; supporting the importance of pre-hospital care and the implementation of early resuscitation techniques.

In the present study, intact males were more likely to sustain trauma and more like to be non-survivors than neutered males. In civilian dogs, neutered males were also found to be more likely to survive their injuries than intact male dogs (35). While this is an interesting correlation, there are likely different reasons for the similar outcomes. The majority of MWDs are not neutered and typically neutering in male MWDs only occurs to treat a medical condition. And as a result, a disproportionate number of MWDs in this study were intact males potentially leading to a type 1 error. In civilian dogs, the reasons for the improved survival in neutered dogs is unclear but has been postulated to be related to the socioeconomic status of the owners (35), a factor not germane to MWDs.

This is the first study to use data from the newly established DoD MWD trauma registry. Until recently, there was no standardized database to report injury data in MWDs, as found with the Joint Theater Trauma Registry for service member injuries (1–3). Previous studies analyzing MWD injuries and illnesses on the battlefield relied on word-of-mouth reporting, massive data calls and screening of archived veterinary medical records available from the DoD Working Dog Center at Lackland Air Force Base (1, 3, 4, 6, 36). For the present study, the DoD MWD trauma registry provided a comprehensive picture of 3 additional MWDs who died from their traumatic injuries as well as 10 MWDs who survived their traumatic injuries. Similar to both the American College of Veterinary Emergency Critical Care (ACVECC) Veterinary Committee on Trauma (VetCOT) trauma registry and the Joint Trauma System registry, the DoD MWD trauma registry is poised to contribute significantly to the care and understanding of MWD injuries as it matures (37, 38).

There are several limitations to this study. First, the sample size was small and only included data for 33 MWD deaths. The vast majority of MWDs who died from trauma had insufficient documentation in their veterinary treatment records to determine the pathophysiology that led to their death. This significantly limited the number of MWDs included in the dataset. Despite this limitation, all available injury data documented in the veterinary treatment record, including after-action reports and evacuation logs, were used to best classify the cause of death in MWDs for inclusion into the study population and better understand the types of injuries MWDs experience. It is expected that the DoD MWD trauma registry will significantly enhance documentation of MWD injuries; so that as more data is accumulated, it will provide a more complete picture of each MWD injury. Second, the dataset relied on the identification of MWDs deaths from various sources, and no comprehensive list of MWD injuries existed until the recent establishment of the DOD MWD trauma registry. As a result, our dataset is not an all-inclusive list of MWD deaths that occurred over a defined period, but rather a sampling of MWD deaths. While efforts were made to include all MWDs who died from their traumatic injuries, the exact number of dogs that died on the battlefield and the total number of dogs deployed during the timeframe for this study is not known. Third, while statistical significance was found in the multivariate and univariate analysis, confidence intervals for these findings were wide, likely as a result of the small sample size. Therefore, the significance of these findings should be interpreted with caution. Another significant limitation of the present study is that the pathophysiologic cause of death was subjectively determined on each MWD by the four authors reviewing the available data (which was often not comprehensive). While the authors have significant experience in both MWD medicine as well as veterinary emergency and critical care, necropsy results were often not available for a definitive cause of death and therefore subjective assessments by the authors were used in this study. There was also a potential selection bias given how MWD cases were identified and included in the study. Finally, it was not possible to compare injuries between survivors and non-survivors with the same MOI due to the low numbers preventing a robust statistical evaluation.

Despite these limitations, this is the first study to date to provide insight into the pathophysiologic causes of traumatic death in MWDs, with hemorrhage and head trauma being most common causes. Most MWDs died of their injuries before reaching veterinary care. To increase the survival of MWDs on the battlefield, further research should focus on developing new interventions and techniques to mitigate the effects of the pathophysiology noted to cause death MWDs (specifically hemorrhage control and traumatic brain injury) and continued efforts to decrease the time from point of injury to veterinary care should be made. Given that non-DVM care was found to be critical for MWD survival, efforts should be continued to train human health care providers and combat medics who often find themselves treating MWDs at both the point of injury and in the prehospital setting. Understanding the causes of death in MWDs is vital to understanding MWD fatalities and is essential to improve in MWD casualty care. Further research is also needed to refine the pathophysiologic causes of death in the MWDs with larger data sets as they become available through the DoD MWD trauma registry.

## Data availability statement

The datasets presented in this article are not readily available because dataset is owned and managed by the US Army Institute of Surgical Research. Requests to access the datasets should be directed to thomas.h.edwards.civ@health.mil.

## Author contributions

AS: Data curation, Methodology, Writing – original draft, Writing – review & editing. TE: Conceptualization, Funding acquisition, Methodology, Project administration, Resources, Supervision, Writing – original draft, Writing – review & editing. CR: Supervision, Writing – original draft, Writing – review & editing. GY: Data curation, Methodology, Writing – original draft, Writing – review & editing. SM: Data curation, Formal analysis, Methodology, Supervision, Validation, Writing – original draft, Writing – review & editing.
